# Prevalence and causes of blood donor deferrals among clients presenting for blood donation in northern Tanzania

**DOI:** 10.1371/journal.pone.0206487

**Published:** 2018-10-25

**Authors:** Donath Mkenda Valerian, Wilhellmuss I. Mauka, Debora Charles Kajeguka, Maseke Mgabo, Abdu Juma, Lelo Baliyima, Geofrey Nimrod Sigalla

**Affiliations:** 1 Faculty of Medicine, Kilimanjaro Christian Medical University College, Moshi, Tanzania; 2 Northern Zone Blood Transfusion Centre, Moshi, Kilimanjaro, Tanzania; 3 Institute of Public Health, Kilimanjaro Christian Medical University College, Moshi, Tanzania; 4 Institute of Rural Development Planning, Dodoma, Tanzania; 5 National Blood Transfusion Services Head office, Dar es Salaam, Tanzania; 6 Department of Health, Evangelical Lutheran Church in Tanzania Headquarters, Arusha, Tanzania; Food and Drug Administration, UNITED STATES

## Abstract

**Introduction:**

Blood is an important requirement in different medical and surgical conditions with half of all donations are from developing countries. Lack of eligibility among blood donors who present for blood transfusion, called blood donor deferral is associated with the unsustainable and inadequate amount of blood collected by blood banks worldwide. However, the prevalence and causes of blood donor deferrals are not well known in Tanzania where less than one-third of actual needs of blood is collected, leading to unmet demand of blood for transfusion, and causing unwanted morbidity and mortality.

**Materials and methods:**

This was a retrospective analysis of blood donors at northern zone blood transfusion center, Tanzania from January to December. 2016. Donor’s data were transferred to Statistical Package for Social Studies (SPSS) program version 20.0 for analysis. Descriptive statistics was used to summarize data and comparisons made by type of donor and deferrals using Chi-square test.

**Results:**

A total of 14377 participants were studied whereby 12775 (88.9%) were voluntary non-remunerated blood donors. The blood donor deferral rate was 12.7% and deferral was significantly more likely in females, with increasing age above 31 years, who came from nearby regions from where the blood bank is located and/or a family replacement donor *(P value <0*.*01)*. Overall, infections contributed to 62% of all deferrals and low hemoglobin was the leading cause of temporary deferrals while Hepatitis B lead the permanent deferral causes.

**Conclusions:**

Blood donor deferral is a significant problem in northern Tanzania and accounts for more than one-tenth of all prospective blood donors. Latent and active infections are the leading cause of blood donor deferrals, a picture that mirrors other low income countries especially those located in sub-Saharan Africa. Results of this study calls for appropriate preventive interventions to address prevalent causes of deferrals such as infections with HIV and HBV to tackle low hemoglobin.

## Introduction

Blood transfusion is a crucial life saving therapy to many who have experienced road accidents, maternal hemorrhage, anemia, different surgical procedures and a number of other medical and surgical conditions [1]. Blood comes from blood donors defined as “persons who donates either whole blood or blood products for transfusion” who provide a global estimate of 112.5 million blood donations yearly [2]. About half of all donations come from developing countries where more than 80% of world’s population lives. World Health Organization (WHO) further provide estimations of nearly nine times greater average blood donations rates in developed countries when compared to developing countries, equivalent to 4.6 donations per 1000 people in developing countries compared to 33.1 donations recorded in developed countries. This brings greater restrain to blood donation needs among the under five year old, who are majority users of blood in developing countries when compared to the needs in developed countries where elderly are the majority users.

Persons donating blood may be voluntary non-remunerated blood donors or replacement donors as required by a member of their own family or community [2]. WHO recommends voluntary non-remunerated blood donors over replacement donors due to the degree of blood safety from the two groups. Although a person can voluntarily decide to donate blood, they may be disqualified from donating blood due to reasons pertaining to the donors’ safety and/or recipient safety, which is simply referred to as donor deferral [3]. Deferral may be temporal postponement or permanent exclusion from donating blood due to being suspected or confirmed of having an infectious disease, hematological disease, or any other medical condition that will either influence the safety of blood or affect donors’ own health [4]. However, the prevalence of blood donor deferrals varies widely, and examples that follow substantiate the variations. In Asia, blood donor deferrals differ from one locality to another [5] and different studies report the prevalence that ranges from 4.6 to 30% [6–8]. Observation in different countries within Europe shows that the prevalence of blood donor deferrals is slightly lower that from Asia [9,10]. In Africa, the prevalence of blood donor deferrals seems to be comparable to that of middle income countries. For example, the prevalence is 10.8% in Ivory Coast [11], and 7% in Zimbabwe [12]. These studies confirm that blood donor deferral is an issue in all countries including Tanzania however, the prevalence is not known. Regardless of the prevalence of deferral, we may generally note that it is an issue of concern to most blood transfusion centers in the world; it affects both low income and high income countries and leads to inadequate blood for transfusion due to lack of eligible blood donors [5]. Blood donor deferral is a sad experience to the donor and the blood bank as whole [13].

Analysis of the causes of blood deferrals show that the causes do differ from one country to the other, calling for a need of setting specific analysis of causes based on the donor selection criteria. However, low haemoglobin has remained to be the main cause of temporal deferral in many places. For example, in Turkey the leading cause of donor deferral is low haemoglobin 20.7% followed by common cold and/or sore throat 17.7%, high risk sexual activities 6.7%, hypertension 5.6% and polycythaemia 2.8% [9]. Similar findings on the role of hemoglobin of deferral has been reported in Netherlands [14] and Asian countries [13]. However, risk factors related to infections of Human Immunodeficiency Virus (HIV) and Hepatitis B have been reported to be the leading cause of permanent blood deferrals in other studies [7,15]. Understanding causes of blood donor deferrals are important in instituting appropriate preventive strategies towards conditions identified, including appropriate referral systems for clinical care.

According to WHO annual blood estimates formula, Tanzania needs 508,000 units of blood annually, but the amount of blood collected in 2016 was less than two-fifths of actual needs (196,735 units) [16]. About 71% of the collected blood is used during child birth and by children under five years of age. To make sure that there is constant supply of blood for transfusion in Tanzania, the Tanzania national blood transfusion services (NBTS) was established in 2004. This was followed by establishment of centralized system through the establishment of seven zonal centers. As the part of NBTS policy [17], guidelines [18] to ensure high standards of blood safety, screening and testing for transfusion transmissible infections (TTIs) is done and include infections such as Human Immunodeficiency Virus (HIV), Hepatitis B Virus (HBV), Hepatitis C virus (HCV) and Syphilis. Screening and deferring clients with medical conditions like fever, low hemoglobin, unstable blood pressure and social reasons like risk behaviors such as sex workers and drug users is done. Although there has been a system to screen and document causes of deferral in Tanzania, no studies have presented a comprehensive analysis on the causes of blood donor deferrals in the country. Information on deferral causes for blood transfusion is important when considering future interventions that aim at increasing blood donation and in the prevention of diseases and conditions associated with blood. Increasing demand of blood for transfusion calls for a need to maximize enrollment to blood donation program and increase the number of those who ultimately donate blood. This study was therefore aimed at estimating the prevalence of blood donor deferrals and to identify the causes of deferrals in the northern zone blood centre in Tanzania.

## Materials and methods

### Study design and setting

This was a cross-sectional study where information from all participants who presented for blood donation at the northern zone blood bank from January 1^st^ to December 30^th^ 2016 were retrospectively analyzed between March and June 2017. The northern zone blood bank center is one of the six centers in Tanzania assigned to collect blood from all donors in four regions of Kilimanjaro, Arusha, Tanga and Manyara. The center was established in 2005 and is located in Moshi municipality about four (4) kilometers from Moshi town, just proximal but within the compound of KCMC Hospital–a consultant hospital in the zone. This center deals with blood donor recruitment, blood collection, screening and distribution of blood to all health facilities in the area. The goal of northern zone blood transfusion center was to collect 15,846 blood units in 2016, however it managed to collect 7,163 (45%) blood units [16]. The center serves a total population of about 6,949, 880 people from the four regions, which is 16% of the Tanzanian population. To achieve the goal the center had to collect blood from voluntary non-remunerated blood donors as well as from replacement donors within the zone.

The procedures for donor intake is as follows: The clients presents for blood donation at the center where they are received at reception and registered. This is followed by counseling provided by trained donor counselors. This counseling process goes in hand with checking on the eligibility for blood donation using a standardized donor questionnaire. The questionnaire inquires about the socio-demographic characteristics of participants such as age, marital status, occupation and address; the type of donor–either voluntary or replacement; and general health check of the donor in terms of diseases and risks for acquiring transmissible infections such as HIV and HBV. Voluntary non-remunerated blood donor was defined as a person who gives blood on his or her own free will and receives no payment, either in the form of cash or in kind. After counseling, prospective blood donors then receive measurements for weight, blood pressure and hemoglobin estimation. The facility then does testing on blood samples for HIV, Hepatitis B, Hepatitis C and Syphilis. Clients who do not fulfill the eligibility criteria for donation are then deferred either temporarily or permanently depending on reason for the deferral. Blood donor deferral in this study was therefore defined as the temporally postponement or permanent exclusion from donating blood by a person suspected of having an infection, disease or any other medical condition that will either influence the safety of blood or affect donors’ own health. Blood donation is done after the client has signed informed consent.

Regarding testing for TTIs, the Tanzanian NBTS tests four TTI markers which are HIV, Hepatitis B, hepatitis C and Syphilis. These tests are done using ELISA antigen–Antibody combination with test kits shown in Table 1. For each marker, appropriate screening test (Screening Test # 1) is done with three possible results; either negative, positive or grey zone. Those with negative test results are recorded as negative and can be recalled for next donation. The blood unit with positive or grey zone test results for the initial screening test is discarded and subjected to a duplicate repeat (Screening test # 1 in Duplicate Repeat) and then supplemental test (Supplemental test # 2). The donor with the blood unit testing positive after this duplicate repeat are deferred permanently.

**Table 1:** Tanzania NBTS TTI’s testing algorithm.

Marker	Screening test # 1	Screening test # 1 in Duplicate Repeat among those who test positive or are reactive in grey zone	Supplemental test # 2 among those who test positive or are reactive in grey zone
HIV	Murex HIV Ag/Ab Elisa Test	Murex HIV Ag/Ab Elisa Test	Enzygnost HIV Integral 4 Elisa Test
HBsAg	Murex HBsAg Version 3.0 Elisa Test	Murex HBsAg Version 3.0 Elisa Test	Enzygnost HBsAg 6.0 Elisa Test
HCV	Murex HCV Ag/Ab Combination Elisa Test	Murex HCV Ag/Ab Combination Elisa Test	Monolisa HCV Ag/Ab Ultra Elisa Test
Syphilis	Espline TP Syphilis Rapid Test	Espline TP Syphilis Rapid Test	Serodia TPPA Qualitative Test

### Study population and data collection

We were provided by the NBTS with information from all clients who presented for blood transfusion at the northern zone blood bank center from January to December 2016. This study extracted data from all registered clients with complete information on donor type, decision to donate or defer and clearly indicated reason for deferral among deferred clients. Participants excluded from this analysis were those who decided not to proceed with the assessment for qualifying to donate blood before being administered with donor questionnaire. Information extracted from the database included those from donor questionnaire and from laboratory where the results for TTIs and other estimations such as of hemoglobin are documented. Information also included the final verdict of donate or defer.

### Statistical analysis

Statistical Package for Social Studies (SPSS) program version 20.0 was used to analyze the data. Descriptive statistics was used to summarize data where frequency tables and cross tabulation were made while describing the data in numbers and percentages. The association between donor types; voluntary and replacement; and between deferrals and accepted donors, are made using Pearson Chi-square test for categorical variables. Results of the difference were considered statistically significant when p-value was less than 0.05.

### Ethical consideration

The ethical approval for carrying out this study was obtained from the Kilimanjaro Christian Medical University College Research and Ethical Committee (CREC) and the National Blood Transfusion Services Ethical Committee. The letter to request permission to do the research and to access the database for the registered blood donors was granted by the National Blood Transfusion Services. The head of laboratory records, head of blood donors and head of records were informed about the study. The information obtained was used for research purpose only. Confidentiality of participant information was observed during the entire period of the study.

## Results

Out of all clients 16537 presented for blood donation in northern blood transfusion centre, 14377 (87.0%) met inclusion criteria. Reasons for exclusion were incomplete data for the status of deferral, donation and reasons for deferral of participants.

### Demographic characteristics of participants

Demographic characteristics of all participants are presented in Table 2 below. The majority of participant were males, who formed more than three-quarters 11377 (79.1%) of all participants. More than two third of all participants 9719 (67.6%) were aged between15 and 30 years followed by 3377 (23.5%) who were aged 31 to 45 years. Almost half of the participants 6671 (46.4%) came from Kilimanjaro region where the center for blood transfusion in the northern zone is located and nearly a quarter 3408 (23.7%) came from a nearby region of Arusha. Almost nine out of every ten participants 12775 (88.9%) who presented for blood donation were voluntary non remunerated blood donors. Family replacement donors were significantly more likely to be females, with increasing age above 31 years and come from nearby regions of Arusha and Kilimanjaro (P value <0.01).

**Table 2:** Demographic characteristics of participants who presented for blood donation and type of participants (n = 14377).

Variable	Total number of blood donors		Voluntary non-remunerated donors		Family replacement donors		p-value^1^
	n	%	n	%	n	%	
**Sex**							
Male	11377	79.1	10013	88.0	1364	12.0	<0.001
Female	3000	20.9	2762	92.1	238	7.9	
**Age group**							
18–30 years	9719	67.6	9039	93.0	680	7.0	<0.001
31–45 years	3377	23.5	2729	80.8	648	19.2	
46–65 years	1281	8.9	1007	78.6	274	21.4	
**Region address**							
Kilimanjaro	6671	46.4	5333	79.9	1338	20.1	<0.001
Arusha	3408	23.7	3210	94.2	198	5.8	
Manyara	2111	14.7	2069	98.0	42	2.0	
Tanga	2187	15.2	2163	98.9	24	1.1	
**Total**	**14377**	**100**	**12775**	**88.9**	**1602**	**11.1**	

^1^Results of chi-square test that show statistical difference of type of donors

### Prevalence of blood donor deferrals

Out of 14377 participants who presented for blood donation in 2016, 1829 (12.7%) were deferred. About 15.6% of all men were deferred and 12.0% of all female were deferred (Table 3). Blood donor deferrals were also categorized according to age where participants aged 46–65 years had higher deferral rate (27.6%) when compared to those who were in the younger age groups of 18 to 30 years (12.1%) and between 31 and 45 years (17.6%). Replacement donors had higher deferral rate 15.8% as compared to voluntary non-remunerated blood donors who had 12.3%. Deferral was significantly more likely among females, increasing age, came from Tanga or surrounding region of Kilimanjaro and/or a family replacement donor (P value <0.01).

**Table 3:** Characteristics of participants who were accepted or deferred to donate blood (n = 14377).

Variable	Participants who were accepted to donate blood		Participants who were deferred to donate blood		p-value^1^
	n	%	n	%	
**Sex**					
Male	10016	88.0	1361	12.0	<0.01
Female	2532	84.4	468	15.6	
**Age group**					
15–30	8672	87.9	1047	12.1	<0.01
31–45	2872	82.4	505	17.6	
46–75	1004	72.4	277	27.6	
**Region address**					
Kilimanjaro	5979	89.6	692	10.4	<0.01
Arusha	3159	92.7	249	7.3	
Manyara	1935	91.7	176	8.3	
Tanga	1942	88.8	245	11.2	
**Type of donor**					
Voluntary non remunerated	11199	87.7	1576	12.3	<0.01
Replacement	1349	84.2	253	15.8	

^1^Results of chi-square test that show statistical difference of donation status

### Causes of blood donor deferrals

Detailed analysis of permanent and temporary causes of blood donor deferrals is shown in Table 4 below. The general picture show that infections contribute to 62% of all deferral causes in this study. Looking at temporary causes, slightly more than half of all blood donor deferrals 927 (50.7%) were due to temporary causes. Of all temporary causes, low haemoglobin was the leading cause; contributed one-fifth (21.1%) of all causes of blood donor deferrals and the majority of participants with low haemoglobin were females 224 (58.0%). Syphilis was the second leading cause of temporary blood donor deferral, carrying about one-tenth 171 (9.3%) of the remaining causes. Majority of those who were deferred because of syphilis were males 149 (87.1%). However, the prevalence of syphilis in the study population was 1.2%. Of all permanent deferrals causes, hepatitis B was the leading cause. It contributed more than a quarter (29.6%) of all deferrals. About 90% of those deferred due to hepatitis B were males. HIV was the second leading cause of permanent deferrals accounting for 13.3% of all deferrals. Of all participants who were deferred because of HIV, 81.1% were males. The prevalence of HBV, HIV and HCV in this study was 3.8, 1.7 and 0.1% respectively.

**Table 4:** Frequency distribution of temporary and permanent deferrals causes by sex (n = 1829).

Causes	Sex					Total	
	Male			Female			
	n		%	n	%	n	%
**Temporary Causes**							
Low haemoglobin		162	42.0	224	58.0	386	21.1
Syphilis		149	87.1	22	12.9	171	9.3
On medication		84	79.2	22	20.8	106	5.8
High/low blood pressure		43	66.2	22	33.8	65	3.6
Risk behavior*		25	89.3	3	10.7	28	1.5
Infections**		32	65.3	17	34.7	49	2.7
Others***		25	56.8	19	43.2	44	2.4
Sub total		578	62.4	349	37.6	927	50.7
**Permanent causes**							
Hepatitis B (HBV)		489	90.4	52	9.6	541	29.6
HIV		198	81.1	46	18.9	244	13.3
Hepatitis C (HCV)		31	22.6	106	77.4	137	7.5
HIV and HBV		0	0	15	100	15	0.8
HIV and HCV		0	0	5	100	5	0.3
HBV and Syphilis		0	0	11	100	11	0.6
HCV and Syphilis		0	0	1	100	1	0.1
HIV and Syphilis		0	0	2	100	2	0.1
HIV, HCV and Syphilis		1	100	0	0	1	0.1
HCV and HBV		3	37.5	5	62.5	8	0.4
Others****		9	60	6	40	15	0.8
Sub total		783	86.8	119	13.2	902	49.3
**Total**		1361	74.4	468	25.6	1829	100

* Body piercing Tattoo, Sex with new partner, Risk behaviour, Alcoholism, Prostitution and Intravenous drug use

** Genitourinary tract infections, Parasitic infections, Malaria, Typhoid, High temperature, URTI, Gastro intestinal infections, TB contact, Sexual transmitted infections, Disease of digestive system

*** Injury or toxication, Vaccination, Menstrual period, Donor faint before donation, Major operation, Eating less than 8 hours, Pregnancy in the past 6 month, Other forms of skin cancer, Blood transfusion-Received allogenic, Breast feeding, Traditional cut, Household contact with hepatitis B, Household contact with hepatitis A, Household contact with unknown hepatitis

**** Heart or circulatory disease, Asthma chronic obstructive disease, Asthma and use steroids, Hematological malignancy, Neurological disease, Psychical disease and Epilepsy

## Discussion

This study aimed to determine the prevalence and causes of blood donor deferrals among clients presenting for blood donation in northern zone Tanzania. The prevalence of blood donor deferrals in our study was found to be 12.7%. Analysis of deferral causes indicated that slightly more than half of all deferred clients were due to temporary causes. Infections were the leading cause of deferral in this study by 62%; with Hepatitis B being the leading single cause followed by low haemoglobin and HIV. The majority of those who were deferred were replacement donor when compared to voluntary non-remunerated donors.

Exploring on the background characteristics of the study subjects, the majority of participants were males, with a male to female ratio of 3.8:1. Other studies have indicated very high male to female ratio [3,13,19]. Having very high male to female ratio, as compared to our study has implication to the study results as some of deferral causes are more prevalent in either males or females. For example, having more males in the study decreased the deferral rates in some studies since females are more deferred than males due to low haemoglobin [13,19]. The present study also indicated that voluntary non-remunerated blood donors were nearly 90% of all the blood donors with remaining proportion being replacement donors. Other studies on blood donor deferrals have found a reverse composition where replacement donors are more than voluntary non-remunerated blood donor [7,19]. Voluntary non remunerated blood donors are safer than replacement donor [20]. However, the reported deferral rates by these studies are within similar range with what has been reported in the present study. The characteristics of participants enrolled in these studies therefore influence the deferral rate, calling for further exploration on other factors. This might be due to differences in geographical location where these studies have been done, difference in levels of infections and/or awareness towards blood donor eligibility among populations where these studies were conducted.

In the present study the prevalence of blood donor deferrals was 12.7%. To the best of our knowledge there is no similar study that have been done in Tanzania thus no other study in the country to be compared with the present study. The deferral rate reported in our study is comparable with what has been reported in other studies done elsewhere. While some studies have shown congruent prevalence results with the present study [21,22], some have reported slightlyr lower [11,13,19] or higher prevalence rates of deferral [7,9,15]. The studies which reported congruent prevalence results used the same study design and tools, and were from low income countries. Most of these studies used donor screening questionnaire together with TTIs result from laboratory and had employed retrospective study design. In that regard, all the studies presenting comparable results with the present study assessed the pre-donation and post-donation deferrals. Of the studies that reported lower rates of deferral [11,13,19], two studies used information on TTI’s [11,19] similar to the methodology of the present study. Similarly, two of the studies which reported higher rates of deferral than what is reported in the present study included results of TTIs in their analysis [7,15]. It can therefore be concluded that the variation in deferral rate presented in these studies when compared to the present study might be explained by the difference in donor eligibility criteria, level of awareness of the population from where donors come on blood donor eligibility criteria and social economic status due to difference in geographical location. In middle and high income countries the level of awareness on blood donor criteria is high, have good nutrition status and low prevalence of infectious diseases [23] which results in the prevalence of deferrals to be low. More strict blood donor eligibility criteria and the level of keenness of blood donor counselors on exploring risk behaviors to clients may lead to the increased prevalence of deferrals. The differences in social-economic status between the countries also contribute to differences in the prevalence of deferrals. For example, with high prevalence of low hemoglobin in low income countries when compared to high income countries [24], low income countries tend to have high prevalence rates of deferrals.

The common cause of blood donor deferrals in our study was infections, which are measured by the positivity of TTIs when screening, accounted for two third of all deferrals. TTIs that are measured in northern zone blood transfusion center are HBV, HCV, HIV, and Syphilis. These results correspond to the nature of low income countries where there is a double burden of infectious diseases and non communicable diseases [25]. Prevalence of infectious and non communicable disease plus malnutrition is higher when compared to the high income countries [26]. The present study further found that HBV was the leading cause of permanent deferrals followed by HIV. Other studies elsewhere have indicates similar trend [5,22,27]. Our study has found that HBV is more prevalent among blood donors (3.8%) than HIV (1.7). Even in the general population of Tanzanians, the prevalence of HBV (6.0%) [28] is higher than that of HIV(4.7%) [29]. It is important noting here that clients testing positive for HIV and HBV will never donate blood, reducing the pool of potential donors in the general population. However there is low level of awareness on HBV, its mode of transmission, its causative agent and its consequences among the general population when compared to HIV in the country [30]. This might be the reason as to why the prevalence of HBV is high among the clients who presented for blood donation in northern zone blood transfusion center. If those positive on HBV and HIV are refereed to appropriate services then Blood Transfusion centers will have identified the diseases and referred to appropriate care, improving the integration with other health care services. The Health Sector Strategic Plan for the health sector and the national policy on health emphasizes the need for continuous care and highly recommends referrals to appropriate care [31]. However, there is a need to balance between the risks for test seeking behavior among blood donors which may attract high risk persons to donate blood and increase the likelihood of window period donations. The best practice is improving community based initiatives for screening of infections coupled with preventive messages, while linking those tested positive for treatment in nearby centers.

The present study has shown that low hemoglobin was the leading temporary cause of blood donor deferrals and the second leading cause of all deferrals followed by Syphilis. This study implies that low hemoglobin is prevalent in northern zone. Similar finding as in our study was reported in Turkey [9], Netherlands [14], Asian countries [13] and in other parts of the world [32,33]. The low Hb is caused by parasitic infections like hookworm and poor nutrition status like low consumption of iron containing diet, Vitamin B12 and folic acid [34]. It seems that most of the participants don’t know about their health status as Hb estimation is a simple and cheap measure of which at least many could have known before presenting at the blood transfusion center. Some of those with low Hb could have known their status before they presented themselves for blood donation. This may mean that the level of understanding on blood donation eligibility criteria is very low among population in the northern zone. From public health perspective, low hemoglobin is amenable to address in the short term and depending on the root cause, re-entry programs to donation are important among clients deferred because of low Hb levels.

The rate of blood donor deferrals in the present study was higher among replacemenr donors than in voluntary non remunerated blood donors. Similar findings have been documented by Stokx et al and by Meinia and Sawhney where replacement donors were deferred more than voluntary non remunerated blood donors [13,35]. This imply that voluntary non remunerated blood donors are relatively safer than replacemnt donors, inline with the WHO recommendation [36]. Regarding the proportion of type of donors, there has been ethusiasm among blood transfusion centers in order to comply with WHO recommendation which requre 100% of all blood to be donated from voluntary non remunerated blood donors [36]. Some of blood donation settings have been reported to falsify the donor status from replacement donors to voluntary donors. We may not know if this may have happened in our setting where 90% of the participant were voluntary non remunerated blood donors.

When interpreting the result of this study, it is important to cosider some of the study limitations. A considerable number of participants had incomplete information and were excluded from this study. Due to having incomplete information, we were not able to compare with those we included in the study. Also the study could not capture the information from self defferals blood donors (who included below 18 years and underweight 50kg), who were not registered into blood donor database at transfusion centre. The potential donors who declined interview and were not recorded may constitute special risk factors different from those who accepted interview. We may therefore not know their influence to the results presented in study. Regardless of these limitation, the present study had several strengths. The study has used a very large sample size. The study also present the combined information from blood donor screening questionnaire and TTIs results thus studying both pre-donation and postdonation deferral causes at the same time.

## Conclusion

A siginificant proportion of blood donor deferrals has been reported in the present study, that accounted for the inadequate supply of blood for transfusion. Transfusion transmissible infections are the leading cause of permanent blood doonor deferrals where HBV and HIV forms the highest proportion of infections. Low Hb is a leading cause for temporary deferal. There is a need to improve blood donor recruitment plans by increasing awareness of the people on blood donation and the causes for deferrals. Mass education on HBV is important so as to increase awareness of the population and ultimate prevention of HBV.
